# Feasibility of a Percutaneous and Non-Fluoroscopic Procedure for Transcatheter Mitral Valve Edge-to-Edge Repair

**DOI:** 10.31083/j.rcm2412346

**Published:** 2023-12-12

**Authors:** Shuyi Feng, Pengxu Kong, Shouzheng Wang, Fujian Duan, Fengwen Zhang, Yongquan Xie, Zefu Li, Wenchao Li, Xiangbin Pan

**Affiliations:** ^1^Department of Structural Heart Disease, National Center for Cardiovascular Disease, China & State Key Laboratory of Cardiovascular Disease, Fuwai Hospital, Chinese Academy of Medical Sciences & Peking Union Medical College, 100037 Beijing, China; ^2^National Health Commission Key Laboratory of Cardiovascular Regeneration Medicine, National Clinical Research Center for Cardiovascular Diseases, 100037 Beijing, China; ^3^Department of Anesthesiology, State Key Laboratory of Cardiovascular Disease, Fuwai Hospital, Chinese Academy of Medical Sciences & Peking Union Medical College, 100037 Beijing, China; ^4^Department of Pediatric Cardiac Surgery, Huazhong Fuwai Hospital, Henan Provincial People’s Hospital, Zhengzhou University People’s Hospital, 451464 Zhengzhou, Henan, China

**Keywords:** mitral regurgitation, transcatheter mitral valve repair, MitraClip, echocardiography, interventional therapy

## Abstract

**Background:**

Transcatheter edge-to-edge repair (TEER) of the mitral valve has emerged as an alternative treatment for mitral regurgitation (MR). However, the high radiation exposure during the process has been associated with multiple adverse effects for medical staff. In this study, we assessed the feasibility and safety of TEER performed solely under the echocardiographic (echo) guidance.

**Methods:**

Between April 2021 to August 2021, we retrospectively collected characteristics of 23 patients with MR who underwent TEER under echocardiographic guidance exclusively. Follow-up evaluations were performed at 1- , 3-months and 1-year post procedure.

**Results:**

All 23 patients (mean age, 66.1 ± 12.1 years; 65.2% males) successfully underwent echo-guided TEER, with 22 patients under transesophageal echo (TEE) guidance and 1 patient under transthoracic echo (TTE) guidance for severe esophageal stenosis. Of the patients, 60.9% received 1 implant and 39.1% received 2 implants. The median total procedural time was 130 (interquartile range, IQR: 90–150) min and the device procedure time was 73 (IQR: 58–100) min. The median length of stay was 6 (IQR: 5–9) days. At 3-months follow-up, 63.6% of patients had an MR ≤1+ and 90.9% had an MR ≤2+ (*p* < 0.001 vs. baseline). Improvement in functional status was observed, with 40.9% of patients classified as New York Heart Association (NYHA) functional class I and 45.5% as NYHA functional class II (*p* < 0.001 compared to baseline) at 3-months. At 1-year follow-up, 90.4% maintained MR reduction with MR ≤2+ (*p* < 0.001 vs. baseline). Single leaflet device attachment (SLDA) occurred in one patient (4.3%) 1-week post procedure.

**Conclusions:**

This retrospective, single-center, and pilot study demonstrates the feasibility, safety, and low complication rates of TEER guided solely by echocardiography. Our findings support the systematic use of echocardiography as the sole guidance modality for TEER, highlighting its potential as an alternative to fluoroscopy-guided procedures. Further multicenter and comparative studies are warranted to confirm these results and provide a more comprehensive evaluation of this approach.

## 1. Introduction

Mitral regurgitation (MR) is one of the most prevalent valvular heart diseases and is associated with increased morbidity and mortality [1]. During the past decade, transcatheter edge-to-edge repair (TEER) of the mitral valve has emerged as an alternative for high-risk patients.

TEER utilizes both transesophageal echocardiography (TEE) and fluoroscopy for guidance. However, long-term exposure to scatter radiation and the necessity of wearing a heavy lead apron have been associated with several adverse effects among medical personnel, including musculoskeletal disorders [2], cataract formation [3], early carotid atherosclerosis [4], reproductive dysfunction [5], and potentially an increased risk of malignant tumors [6]. In addition, fluoroscopy may not be suitable for patients with existing malignant tumors or those who are pregnant, and its use requires expensive medical equipment.

To circumvent the limitations of fluoroscopic guidance, percutaneous and non-fluoroscopic (PAN) procedures have gained significant attention in the management of structural heart diseases. These procedures, which include interventions for conditions such as atrial septal defect, ventricular septal defect, patent ductus arteriosus, aortic and mitral balloon valvuloplasty, utilize TEE or transthoracic echocardiography (TTE) as the sole image guidance [7, 8, 9, 10, 11]. Recently, PAN procedures have been attempted for TEER in a patient with concomitant MR and lung cancer [12]. Here, we aimed to evaluate the safety and effectiveness of this procedure by analyzing a retrospective series of 23 consecutive MR patients who underwent TEER guided solely by echocardiography at our center.

## 2. Methods

### 2.1 Patients

The study was approved by the ethics board of Fuwai Hospital, Chinese Academy of Medical Sciences (No. 2021-1530). All patients and legal guardians signed informed consent for the operation and clinical record review.

This present retrospective, observational, cohort study was conducted at Fuwai Hospital. Data were collected from patients with symptomatic MR who underwent TEER with MitraClip guided solely by TEE or TTE from April 2021 to August 2021. Baseline assessment encompassed demographics, symptoms, comorbidities, routine laboratory testing, risk evaluation, electrocardiogram findings, and TTE results. The heart team, comprising of multidisciplinary experts, evaluated all patients to determine their suitability for TEER according to the current guidelines [13]. Subsequently, patients with contraindications to radiation or contrast agents, such as those with a history of malignant tumors, pregnancy, severe renal failure, or known allergies to contrast agents, were further screened for potential eligibility for PAN procedure via TEER.

### 2.2 Procedure

**Fig. 1.**
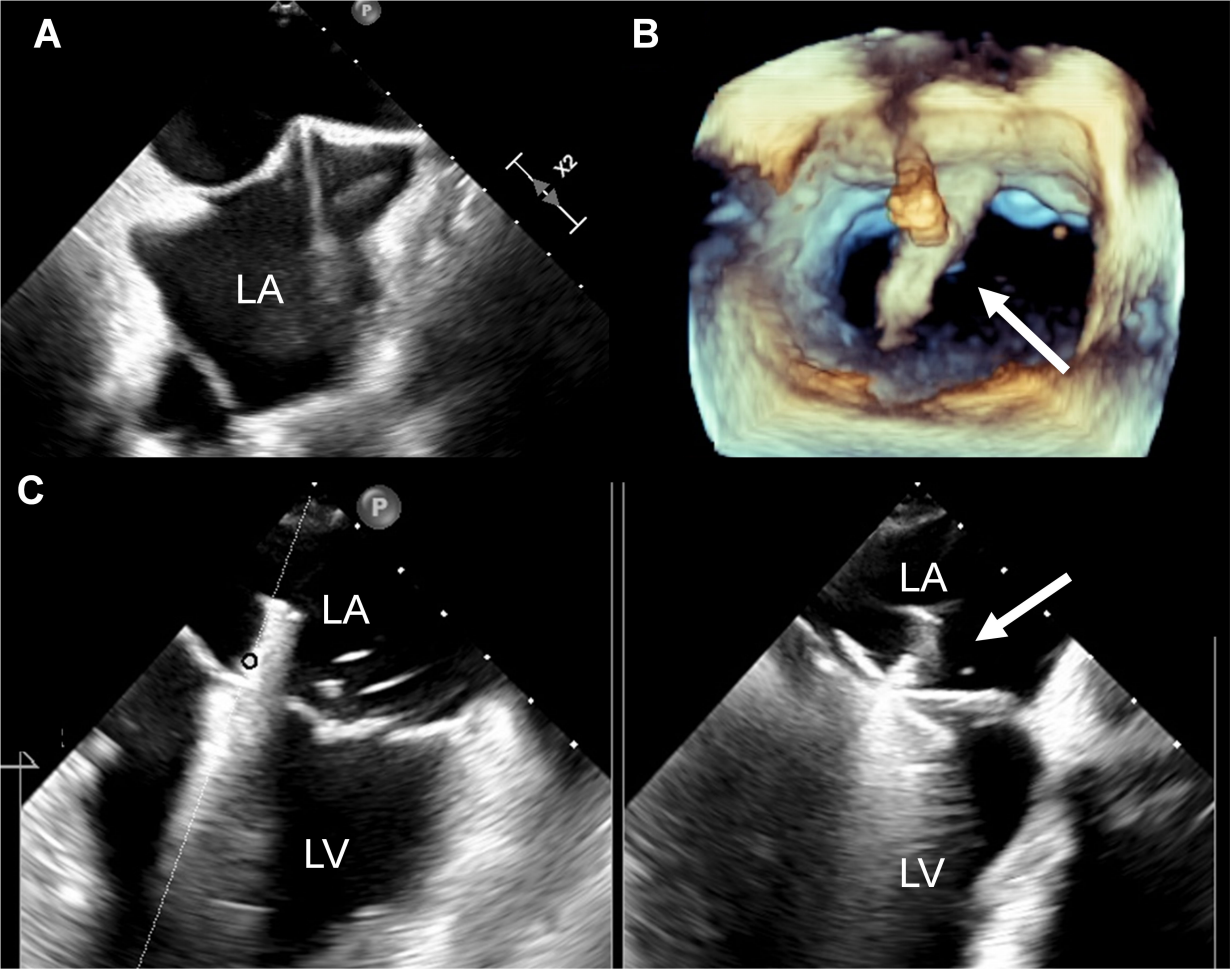
**TEE guided TEER procedures**. (A) Tent-like protrusion on the interatrial septum. (B) 3D echocardiogram showing MitraClip CDS in the left atrium (arrow). (C) TEE showing MitraClip clamping anterior and posterior valve leaflets (arrow). TEE, transesophageal echocardiography; TEER, transcatheter edge-to-edge repair; LA, left atrium; LV, left ventricular; 3D, three-dimensional; CDS, clip delivery system.

All procedures were performed in the operating room under general anesthesia in a supine position. The working distance was determined as the distance from the puncture point to the third intercostal space on the right side of the sternum. A 10 Fr arterial sheath was introduced through femoral vein puncture. An MPA2 catheter and the super-stiff guide wire were inserted according to the working distance. Echocardiography was used to visualize the bi-atrial view at 90 degrees, monitoring the guide wire as it entered the right atrium from the inferior vena cava. The inserted length of the MPA2 catheter was marked after the guide wire reached the atrial septum, allowing for correction of the working distance. The SL1 puncture catheter was inserted at the working distance along the guidewire. The puncture needle was then inserted. By rotating the puncture catheter, a tent-like protrusion was created on the interatrial septum, with the puncture made posterior to the fossa ovalis (Fig. 1A, Video 1). The puncture position was adjusted under ultrasound X-plane guidance. The puncture point was at least 4.0 cm away from the mitral valve annulus. Normal saline was injected to perform contrast-enhanced echocardiography, with microbubbles filling the left atrium to confirm successful trans-septal puncture. The MitraClip clips (CDS0601-XTR or CDS0601-NTR, Abbott, Chicago, IL, USA) were pre-installed. The clip delivery system (CDS) with the working distance mark was introduced into the guide catheter. For navigating to the annulus, a three-dimensional (3D)-view was utilized to visualize the structure of the left atrium. Then the CDS was oriented perpendicularly to the long axis of the leaflet edges under the 3D-view guidance (Fig. 1B, Video 2). Meanwhile, the bi-commissural view was also used to monitor the direction of the CDS. Then, the clip was advanced into the left ventricle just below the mitral leaflet edges, as visualized by the left ventricular (LV) outflow tract and apical two-chamber view. Leaflet grasping, leaflet insertion, and MR assessment were performed in a standard fashion (Fig. 1C). Deployment of more than one MitraClip device was allowed if necessary.

**Video 1.** **The embedded movie may also be viewed at https://doi.org/10.31083/j.rcm2412346**.

**Video 2.** **The embedded movie may also be viewed at https://doi.org/10.31083/j.rcm2412346**.

All patients were admitted to the intensive care unit (ICU) for monitoring and treatment after the procedure. Aspirin therapy was initiated on the second day after the procedure and continued for 6-months.

### 2.3 Outcome Measures

The operation success rate was defined as the proportion of cases in which the MitraClip device(s) were successfully delivered and deployed. Total procedure time was defined as the duration from anesthesia induction to the removal of the last sheath. Device procedure release time was defined as the time from the initiation of the transseptal procedure to the removal of the clip delivery system.

### 2.4 Follow-Up

Follow-up assessments of clinical outcomes and transthoracic echocardiography were conducted during outpatient visits or through phone interviews at 1-, 3-months and 1-year. Cardiac events were carefully recorded.

### 2.5 Statistical Analysis

Continuous variables are presented as means ± standard deviation, while categorical variables as expressed as percentages. A paired Student’s *t*-test was utilized to compare specific time point with baseline for continuous variables, while the Wilcoxon signed rank test was employed for categorical variables. Statistical analysis was conducted using SPSS 25.0 for Windows (SPSS Inc., Chicago, IL, USA). A *p* value of less than 0.05 was considered statistically significant.

## 3. Results

### 3.1 Patients and Procedures

Between April 2021 and August 2021, a total of 23 patients successfully underwent TEER with exclusive echo guidance at the Fuwai Hospital. Baseline characteristics are summarized in Table 1. The mean age was 66.1 ± 12.1 years, with 65.2% being males, and the mean body mass index (BMI) was 24.0. All patients had moderate-to-severe (grade 3+ or 4+) MR, with 91.3% of patients classified as New York Heart Association (NYHA) functional class III or IV. Based on etiologic classification, 7 patients (30.4%) had functional MR (FMR) and 16 (69.5%) had degenerative MR (DMR). Among the patients, 10 (43.5%) had a single A2/P2 lesion, while the remaining patients presented with A1/P1 or A3/P3 lesions. Two patients (8.7%) underwent MitraClip implantation with the support of an intra-aortic balloon pump (IABP) and 4 patients (17.4%) had a history of malignant tumor. Additionally, 14 (60.9%) patients had severe renal failure, and 5 (21.7%) patients had allergies that necessitated the avoidance of contrast agents.

**Table 1.** **Baseline Characteristics**.

		Overall (n = 23)
Age (yrs)		66.1 ± 12.1
Male		15 (65.2)
BMI		24.0 ± 3.4
Comorbidities		
	Coronary heart disease	8 (34.8)
	Previous percutaneous coronary intervention	7 (30.4)
	Pulmonary hypertension	13 (56.5)
	Atrial fibrillation	4 (17.4)
	Diabetes mellitus	3 (13)
	Malignant tumor	4 (17.4)
	Renal failure	14 (60.9)
	Allergy to contrast agent	5 (21.7)
	Previous heart surgery	3 (13)
	With IABP	2 (8.7)
LVEF (%)		59.4 ± 11.7
NYHA functional class		
	II	2 (8.7)
	III	18 (78.3)
	IV	3 (13)
MR severity		
	3+	11 (47.8)
	4+	12 (52.2)
Lesion region		
	Single A2/P2 segment	10 (43.5)
	Single A1/P1 or A1/P1 combined A2/P2	5 (21.7)
	Single A3/P3 or A3/P3 combined A2/P2	8 (34.8)
Vena contracta width (mm)		7.3 ± 1.3

Values are mean ± SD, or n (%). LVEF, left ventricle ejection fraction; NYHA, New York Heart Association; MR, mitral regurgitation; BMI, body mass index; IABP, intra-aortic balloon pump.

Successful implantation was achieved in all patients, with a mean of 1.4 clips per patient. Among them, 60.9% received 1 implant, while 39.1% received 2 implants. Immediately after the procedure, only 2 patients (8.7%) had residual mild-to-moderate (2+) MR, while the remaining patients demonstrated significant improvement, with MR decreasing to 0+ or 1+ (Table 2). The median total procedural time was 130 (interquartile range, IQR: 90–150) min and the device procedure time was 73 (IQR: 58–100) min. The median length of stay was 6 (IQR: 5–9) days. No complications, such as vascular injury, cardiac tamponade, or MitraClip embolism, were observed in any of the patients.

**Table 2.** **Procedural outcomes**.

		Overall (n = 23)
Successful implantation		23 (100)
Number of clips implanted		1.4 ± 0.50
Total procedure time (min)		130 (90–150)
Device procedure time (min)		73 (58–100)
Postprocedural MR severity		
	0+	9 (39.1)
	1+	12 (52.2)
	2+	2 (8.7)
Length of stay (days)		6 (5–9)

Values are mean ± SD, median (interquartile range) or n (%). MR, mitral regurgitation.

### 3.2 TTE Guided Procedure 

**Fig. 2.**
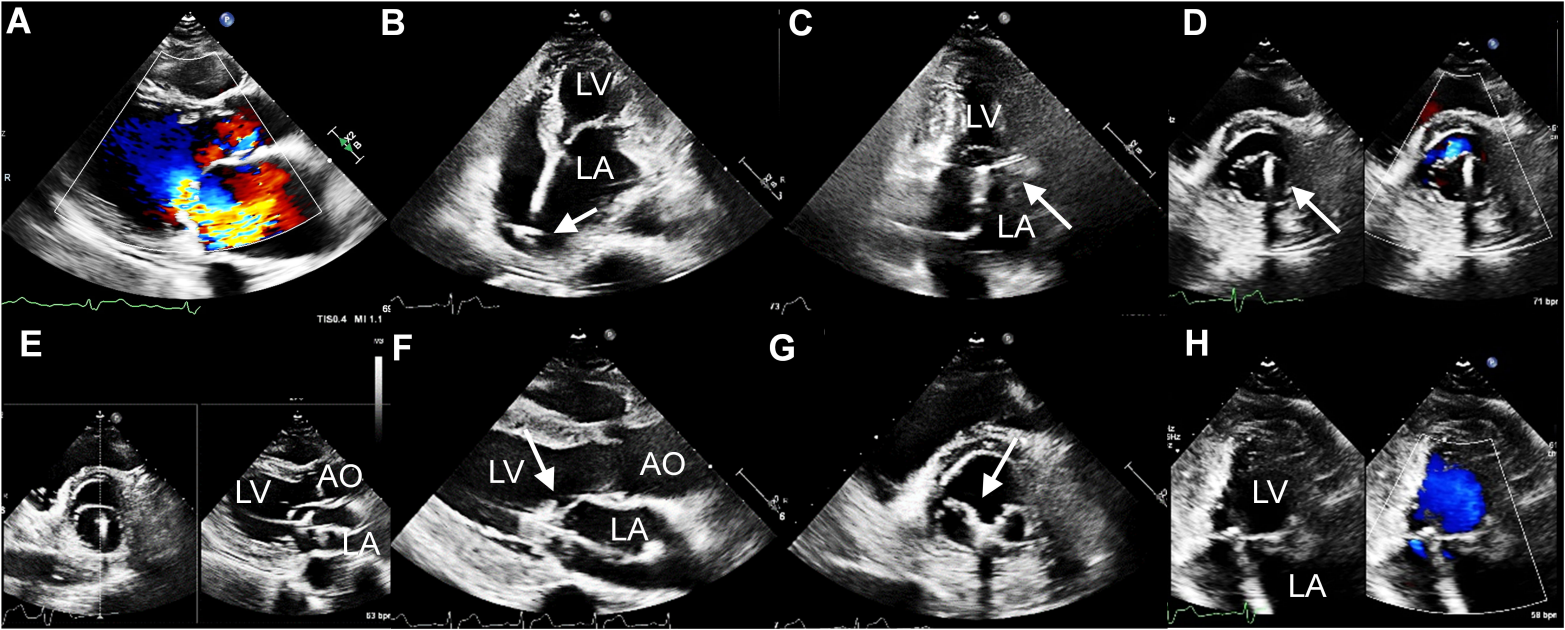
**TTE guided TEER procedure**. (A) TTE showed severe MR. (B) The transseptal puncture under the bi-atrial view (arrow). (C–E) The clip (arrow) was advanced under the guidance of TTE. (F) The leaflets were captured by the clip (arrow). (G) Confirming the position of the clip (arrow) under short-axis view. (H) Postprocedural TTE showed MR decreased to traced. TTE, transthoracic echocardiography; MR, mitral regurgitation; LA, left atrium; LV, left ventricle; AO, aorta; TEER, transcatheter edge-to-edge repair.

One patient with posterior mitral valve leaflet clefts and severe esophageal stenosis was successfully treated under TTE guidance alone (Fig. 2A). The procedure was conducted under general anesthesia. The transseptal puncture was performed posterior to the fossa ovalis under the bi-atrial view (Fig. 2B). Subsequently, the pre-installed MitraClip (CDS0601-XTR, Abbott, Chicago, IL, USA) was introduced along the CDS. With TTE guidance, the clip was advanced to the center of the mitral orifice under the inter-commissural view and short-axis view (Fig. 2C,D, Video 3). The arms of the clip were oriented perpendicularly to the long axis of the leaflet edges (Fig. 2E). Then, the clip was advanced into the left ventricle and pulled back until the mitral leaflets were captured (Fig. 2F). Clip orientation was confirmed again at the short-axis view (Fig. 2G, Video 4). Then the device was closed gradually to optimize the reduction of MR. The MR decreased to trace (Fig. 2H) with a mean mitral valve (MV) pressure gradient of 1.9 mmHg. The total procedure time of TTE guidance was 88 min and the device procedure time was 66 min.

**Video 3.** **The embedded movie may also be viewed at https://doi.org/10.31083/j.rcm2412346**.

**Video 4.** **The embedded movie may also be viewed at https://doi.org/10.31083/j.rcm2412346**.

### 3.3 Echocardiographic Results

**Fig. 3.**
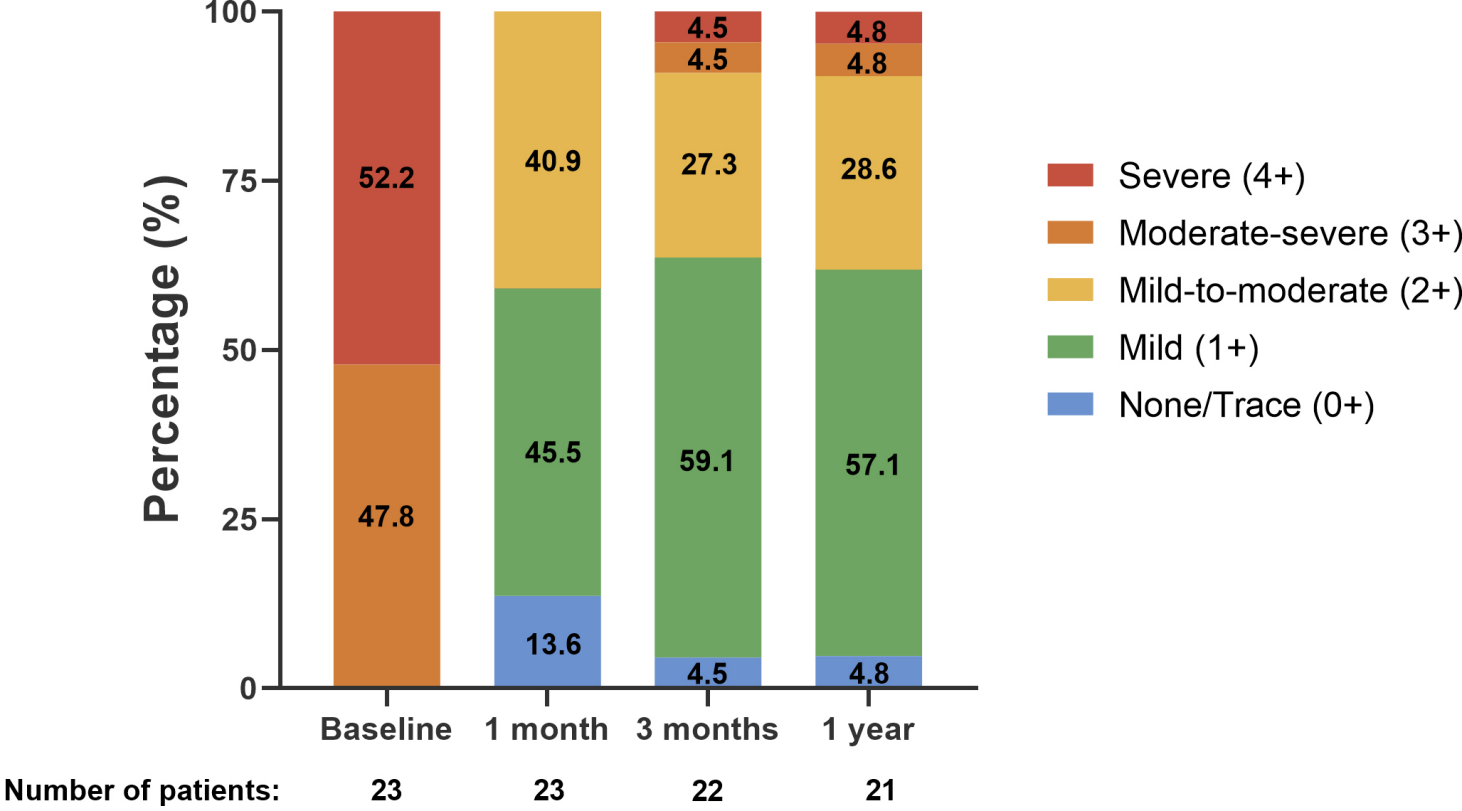
**Change in mitral regurgitation grade up to 1-year follow-up**.

The changes in MR severity as assessed by echocardiography at baseline, 1-, 3-months, and 1-year are shown in Fig. 3. At 1-month, 59.1% of patients had MR ≤1+, and 100% of patients had MR ≤2+, indicating a significant improvement compared to baseline (*p* < 0.001 vs. baseline). Among the 22 patients followed up at 3-months, 63.6% had MR ≤1+, and 90.9% had MR ≤2+ (*p* < 0.001 vs. baseline). Among the 21 patients with echocardiographic data available at 1-year, 90.4% had sustained MR reduction with MR ≤2+ (*p* < 0.001 vs. baseline) (Fig. 3).

Changes in LV volumes, LV ejection fraction (LVEF), and other echocardiographic parameters are shown in Table 3. At 1-month, LV end-diastolic volume decreased from 160.84 ± 43.97 to 124.42 ± 39.24 mL (*p* < 0.001), while LV end-systolic volume numerically but not significantly decreased from 69.81 ± 36.33 to 57.00 ± 27.60 (*p *= 0.028). Furthermore, from baseline to 1-month, significant reductions were observed in LV internal diameter during diastole (from 58.76 ± 5.41 to 51.67 ± 6.34, *p* < 0.001), LV internal diameter during systole (from 39.00 ± 7.85 to 35.20 ± 6.93, *p* = 0.003), and LA diameter (48.73 ± 10.57 to 44.09 ± 11.18 mm, *p* = 0.001). However, LVEF did not show a significant change from baseline to 1-month. At 1-year follow-up, significant reductions of LV end-diastolic volume and LV end-systolic volume were still observed, indicating sustained improvements in LV remodeling (Table 3).

**Table 3.** **1-year echocardiographic outcomes**.

	Baseline	1 month	1 year	*p* value	*p* value
				Baseline vs. 1 month	Baseline vs. 1 year
LA diameter (mm)	48.73 ± 10.57	44.09 ± 11.18	45.48 ± 10.30	0.001	0.155
LV end-diastolic diameter (mm)	58.76 ± 5.41	51.67 ± 6.34	49.47 ± 4.18	<0.001	0.001
LV end-diastolic volume (mL)	160.84 ± 43.97	124.42 ± 39.24	117.93 ± 25.39	<0.001	0.008
LV end-systolic diameter (mm)	39.00 ± 7.85	35.20 ± 6.93	33.93 ± 4.60	0.003	0.017
LV end-systolic volume (mL)	69.81 ± 36.33	57.00 ± 27.60	47.64 ± 18.49	0.028	0.041
LVEF (%)	59.00 ± 11.82	55.00 ± 13.75	61.13 ± 7.99	0.102	0.623

Values are mean ± SD. LA, left atrium; LV, left ventricle; LVEF, left ventricle ejection fraction.

### 3.4 Clinical Outcomes

**Fig. 4.**
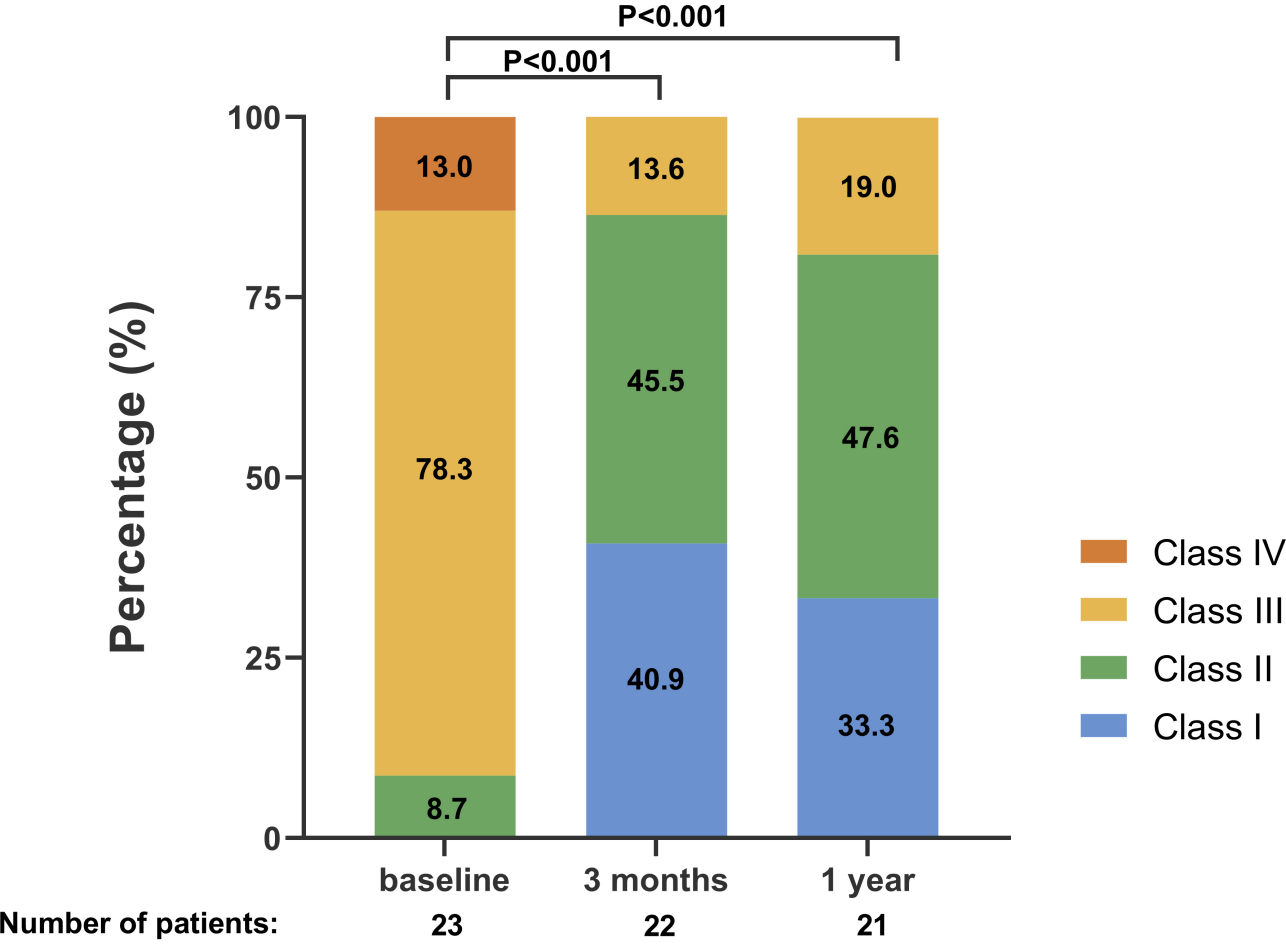
**Change in New York Heart Association functional class at 1-year follow-up**. *p* values were calculated by the Wilcoxon signed rank test for paired patients.

NYHA functional classes at baseline and 3-months are shown in Fig. 4. At 3 months, 40.9% of patients showed an improvement to NYHA functional class I and 45.5% improved to NYHA functional class II (*p* < 0.001 vs. baseline). One patient (4.3%) suffered single leaflet device attachment (SLDA) 1-week after the operation, and subsequently underwent mitral valve replacement surgery 11-days after the operation. At 1-year, 2 (8.7%) patients died, 1 due to heart failure and 1 due to renal failure. However, 80.9% of the patients had improvement to NYHA functional class I/II (*p* < 0.001 vs. baseline) (Fig. 4).

## 4. Discussion

Our single-center experience demonstrates that TEER performed only under echocardiography guidance was feasible, safe, and effective. All patients achieved a reduction of MR to ≤2+ at 1-month, 90.9% maintained the reduction at 3-months, and the reduction persisted to 1-year. Significant improvements in echocardiographic parameters indicated LV reverse remodeling. At 3-months, the NYHA class was significantly improved with 86.4% of patients classified as NYHA class I/II. The echo-guidance-only approach proved to be safe during follow-up with only 1 case of SLDA observed at 1-year follow-up and no reported incidence of device embolization. These results compare favorably with outcomes reported with the traditional procedure performed under fluoroscopy guidance [14, 15, 16, 17].

MR is a common heart valve disease that becomes more prevalent with age [18]. TEER is safe and effective in selected MR patients under both transesophageal echocardiographic and fluoroscopic guidance [19, 20]. McNamara *et al*. [21] suggested that during TEER, interventional echocardiographers received significantly higher radiation doses than interventional cardiologists (median: 10.5 µSv; IQR, 3.1–20.5 µSv vs. 0.9 µSv; IQR, 0.1–12.2 µSv; *p* < 0.001), which raises increased concern for protection, particularly for echocardiographers who stand closer to the source of radiation. Therefore, every precaution needs to be taken to minimize exposure for all member of the cardiac catheterization team [22, 23]. In this context, the PAN procedure might offer potential benefits for the structural heart team, including the operators, interventional echocardiographers, anesthetists, and nurses. On the other side, patients with renal failure or allergies may benefit from avoiding the use of contrast agent. Therefore, we propose that PAN procedures could be encouraged and extended to mitigate the risks associated with radiation exposure. With proper planning and collaboration between interventionists and echocardiographers, the process can be simplified with only TEE or even TTE guidance, while achieving comparable procedural time and results. In addition, the cost-intensive equipment required for fluoroscopic procedures poses a significant financial burden, particularly in underdeveloped areas. PAN procedures, already established for congenital heart disease and valve disease, rely on echocardiography as the only imaging tool [7, 8, 9, 10]. Our previous experience with echo-guided percutaneous treatments has enabled us to successfully perform non-fluoroscopy-guided TEER and expand access to better treatment options for patients with limited medical resources.

The advantages provided by echocardiography include simultaneous and continuous monitoring of mitral valve leaflet movement, confirmation of wire position and direction, and the ability to assess hemodynamic status. Working distance measurement and echo guidance facilitate safe entry of the guidewire and catheter into the atrium. By identifying the tent-like deformation (“tenting”) of the interatrial septum in the four-chamber and short-axis views at the location of the needle tip, echocardiography guidance enables optimal puncture site selection. The position of the clip and mitral valve leaflets can be well visualized by echocardiography, which allows us to adjust the clip direction and clamp location. Residual MR and mean mitral valve gradient can be evaluated by echocardiography immediately after releasing the clip. Using only ultrasound guidance throughout the procedure effectively avoids interference between fluoroscopy and the esophageal probe. The total procedure time (median 130 min) and device procedure time (median 73 min) of our pilot research were comparable to the previous studies performed with fluoroscopy guidance [24, 25, 26]. SLDA occurred in only 1 case. No patients died within 3-month follow-up, and 2 patients died at 1-year follow-up. The frequency of major adverse events was comparable to previous research [24, 26, 27, 28].

TEE is the standard imaging modality used to guide the MitraClip procedure. However, patients who are intolerant to general anesthesia or those with contraindications for TEE have limited treatment options. Previous studies have explored intracardiac echocardiography (ICE) as an adjunctive or sole image guide tool for patients with contraindications to TEE [29, 30, 31, 32]. ICE provides clear intraprocedural imaging under conscious sedation [31]. Here we reported the first TEER case guided by TTE. The total procedure time of this case was 88 min and no severe complications occurred. However, it should be noted that sole TTE guidance during TEER is challenging. First, patients with poor acoustic windows should be excluded from sole TTE guidance. Second, TTE provides limited image information for the septal rim, so the transseptal puncture location (“tenting”) should be carefully evaluated under multiple views by experienced cardiology interventionists and echocardiographers. Third, the CDS should be carefully advanced within the working distance and tracked by echo in the left atrium, to avoid perforating the left atrial appendage. With the advancement of echo images and operative devices, we envision that the TEER by ICE or TTE guidance can become routine in minimalist procedures.

## 5. Limitations

This study has some limitations. First, the retrospective single-center design with a relatively small sample size. Second, the procedures being performed exclusively by experienced investigators. The learning curve of echo-guided procedures by junior doctors with limited experience should be thoroughly evaluated. Third, there was no control group with fluoroscopic guidance, which would better compare the procedural time and complication. Fourth, only 1 case of TTE-guided procedure was included in this study. The safety of this minimalist procedure should be further assessed. Further larger prospective, and comparative studies in more diverse settings with longer-term follow-up are warranted.

## 6. Conclusions

The findings of the present study suggest that that TEER under echocardiography guidance is feasible, safe, and have low complication rates in patients with MR. However, further research is needed, including multicenter studies and comparative investigations with longer-term follow-up, to validate these findings and provide a more comprehensive understanding of the efficacy and safety of echo-guided TEER.

## Data Availability

All data generated or analyzed during this study are included in this published article.
